# Simplified rules-based tool to facilitate the application of up-to-date management recommendations in cardio-oncology

**DOI:** 10.1186/s40959-023-00179-w

**Published:** 2023-10-27

**Authors:** Sherry-Ann Brown, Abdulaziz Hamid, Erin Pederson, Allen Hanna BS, Ragasnehith Maddula, Rachel Goodman, Morgan Lamberg, Pedro Caraballo, Peter Noseworthy, Opeoluwa Lukan, Gift Echefu, Generika Berman, Indrajit Choudhuri, for the Cardio-Oncology Artificial Intelligence Informatics & Precision Equity (CAIPE) and Patient Similarity Algorithms in the Prevention of Cardiovascular Toxicity (PACT) Research Team Investigators

**Affiliations:** 1 Cardio-Oncology Program, Division of Cardiovascular Medicine, Medical College of Wisconsin, Milwaukee, WI USA; 2 Department of Cardiovascular Medicine, Mayo Clinic, Rochester, MN USA; 3 Medical College of Wisconsin, Milwaukee, WI USA; 4 University of Wisconsin - Milwaukee, Milwaukee, WI USA; 5 Department of Medicine, Mayo Clinic, Rochester, MN USA; 6 Department of Internal Medicine, Baton Rouge General Medical Center, Baton Rouge, LA USA; 7 Medical College of Wisconsin, Green Bay, WI USA; 8 Department of Electrophysiology, Froedtert South, Kenosha, WI USA

**Keywords:** Guidelines, Scientific statements, Cardio-oncology, Cancer Survivors

## Abstract

**Background:**

Millions of cancer survivors are at risk of cardiovascular diseases, a leading cause of morbidity and mortality. Tools to potentially facilitate implementation of cardiology guidelines, consensus recommendations, and scientific statements to prevent atherosclerotic cardiovascular disease (ASCVD) and other cardiovascular diseases are limited. Thus, inadequate utilization of cardiovascular medications and imaging is widespread, including significantly lower rates of statin use among cancer survivors for whom statin therapy is indicated.

**Methods:**

In this methodological study, we leveraged published guidelines documents to create a rules-based tool to include guidelines, expert consensus, and medical society scientific statements relevant to point of care cardiovascular disease prevention in the cardiovascular care of cancer survivors. Any overlap, redundancy, or ambiguous recommendations were identified and eliminated across all converted sources of knowledge. The integrity of the tool was assessed with use case examples and review of subsequent care suggestions.

**Results:**

An initial selection of 10 guidelines, expert consensus, and medical society scientific statements was made for this study. Then 7 were kept owing to overlap and revisions in society recommendations over recent years. Extensive formulae were employed to translate the recommendations of 7 selected guidelines into rules and proposed action measures. Patient suitability and care suggestions were assessed for several use case examples.

**Conclusion:**

A simple rules-based application was designed to provide a potential format to deliver critical cardiovascular disease best-practice prevention recommendations at the point of care for cancer survivors. A version of this tool may potentially facilitate implementing these guidelines across clinics, payers, and health systems for preventing cardiovascular diseases in cancer survivors.

**Trial Registration:**

ClinicalTrials.Gov Identifier: NCT05377320.

## Introduction

 Millions of cancer survivors develop cancer therapy-related cardiotoxicity, including atherosclerotic cardiovascular disease (ASCVD) [1, 2]. The number of cancer survivors in the United States is estimated at approximately 17 million [3], and by 2030, that number should rise to more than 22 million [4]. Cardiovascular complications pose a risk to cancer survivors, as they are a leading cause of morbidity and mortality [5–8]. Cancer survivors are more likely to suffer from cardiovascular disease than the general population [5–8]. In racial and ethnic minorities, the rates are even higher [9–13]. Mitigating this risk in all cancer populations remains difficult. Further, the application of recent guidelines, consensus recommendations, and scientific statements to optimize cardiovascular medication and imaging use in this population is limited [14–16]. Additional tools are needed to facilitate the application of these guidelines for the cardiovascular care of cancer survivors.

Cancer survivors continually present to cardiology clinics with adverse cardiovascular effects [17–22]. Cancer survivors, particularly those at high risk for cardiovascular disease, should be identified by cardiologists and oncologists with initiation of cardioprotective measures such as medication and imaging surveillance [14–16]. However, studies have shown suboptimal cardiovascular medication and imaging use in this population [14–16]. Determining an optimal way to facilitate optimal cardiovascular care for cancer survivors, who have a higher risk than the general population of developing cardiovascular disease, remains a challenge. Barriers to the application of these recommendations include limited awareness of these guidelines, as well as lack of guidelines specific to the cancer survivor population for some topics such as ASCVD prevention.

Studies on the utilization of cardiovascular medication use and imaging surveillance in cancer survivors indicate that in particular statins are significantly underutilized in cancer survivors to prevent cardiovascular diseases including ASCVD [14–16]. Indeed, underdiagnosed ASCVD develops in many patients due to cardiotoxicity from pharmacologic and radiation cancer treatments [23]. Many of these patients are therefore lacking evidence-based medical therapy that could prevent ASCVD and other cardiovascular diseases. Notably, the cancer survivor population lacks ASCVD prevention guidelines that are specific to their care. Thus, these patients often do not receive evidence-based medical therapy to prevent ASCVD and address current ASCVD risk despite related guidelines developed for the general population without cancer [16].

In this original study, we hypothesized that a rules-based tool could be created to incorporate crucial guidelines, expert consensus, and medical society scientific statements relevant to the direct cardiovascular care of cancer survivors. We anticipate that developing such a tool could potentially help facilitate the application of more optimal cardiovascular care of cancer survivors.

## Methods

### Study design

**Fig. 1:**
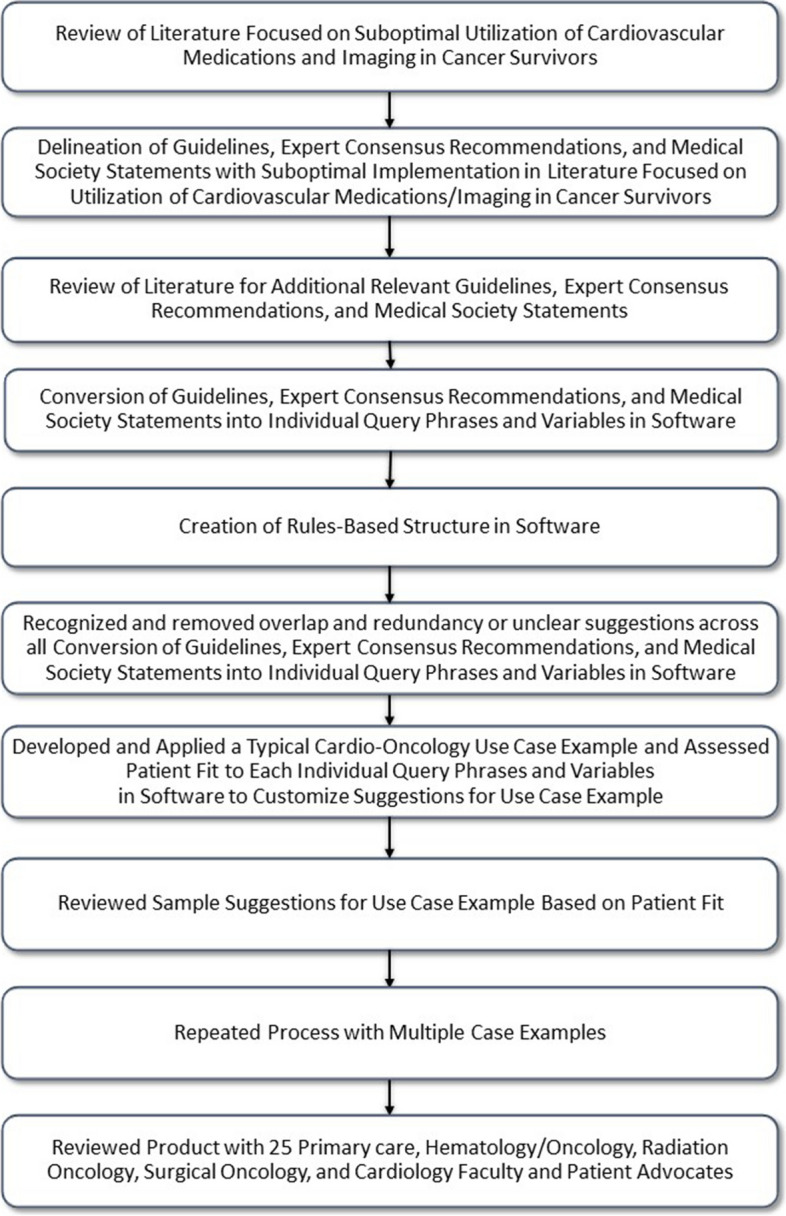
Study Design Flow Chart for Developing Rules-Based Tool

The Medical College of Wisconsin Internal Review Board approved this study. First, we reviewed the literature to determine the extent to which cardiovascular risk and prevention guidelines are being applied in cancer survivors (Fig. 1). We based this on published reports of medication use and imaging surveillance in this population [14–16]. Second, we identified crucial guidelines relevant to the cardiovascular care of cancer survivors (Table 1). We ensured the inclusion of guidelines addressing the gaps noted in medication use and imaging surveillance in this population, based on the lack noted in published reports [14–16]. We then converted these guidelines into query variables and phrases in Microsoft Excel (Microsoft Corporation, Washington, United States), the software used for this work. Accordingly, we created a rules-based structure for applying these guidelines in the software. We then recognized and removed all overlap and redundancy or unclear suggestions across all converted guidelines, expert consensus recommendations, and medical society statements. We then developed and applied a typical cardio-oncology use case example and assessed patient fit to each individual query phrase and variable in the software, to customize suggestions for the use case example. Sample suggestions for the use case example based on patient fit were then reviewed. This process was repeated for 50 additional case examples (see summary of the types of information used for these sample cases in Table 2). The produfct was then reviewed with 25 primary care, hematology/oncology, radiation oncology, surgical oncology, and cardiology clinicians and patient advocates

**Table 1:** Guideline, Expert Consensus, and Scientific Statements in Rules-Based Tool for Prevention of Cardiovascular Disease in Cancer Survivors

Guideline, Expert Consensus, or Scientific Statement
Prevention and Monitoring for Cardiac Dysfunction - ASCO 2017 [24]
Multimodality Imaging Evaluation of Adult Patients during and after Cancer Therapy - ASE/EACVI 2014 [25]
Cardiovascular Screening for Patients on Chemotherapy/Radiation Therapy - SCAI 2016 [26]
Expert Consensus for Multi-Modality Imaging Evaluation of Cardiovascular Complications of Radiotherapy - ASE-EACVI 2013 [27]
Cardiovascular Manifestations From Therapeutic Radiation - JACC 2021 [28]
Guideline on the Primary Prevention of Cardiovascular Disease - ACC/AHA 2019 [29]
Cholesterol Clinical Practice Guidelines - AHA/ACC 2018 [30]

*ACC *American College of Cardiology, *AHA *American Heart Association, *ASCO *American Society of Clinical Oncology, *ASE *American Society of Echocardiography, *EACVI *European Association of Cardiovascular Imaging, *JACC *Journal of the American College of Cardiology, *SCAI *Society for Cardiovascular Angiography and Interventions

**Table 2:** Summary of Types of Information Used for 50 Additional Sample Patients to Test Simple Rules-Based Tool

Variable Categories	Variable Examples
Patient Characteristics	Age, gender, body mass index, exercise level, race/ethnicity, sexual activity
Cardiovascular Risk Factors	Hypertension, dyslipidemia, diabetes, smoking, obesity
Quantitative Markers	Vital capacity, ejection fraction, left ventricular ejection fraction, coronary artery calcium score, QRS duration, QT interval, B-type natriuretic peptide, erythrocyte sedimentation rate, total cholesterol, high density lipoprotein cholesterol, low density lipoprotein cholesterol, fasting triglycerides, hemoglobin A1C, glucose, systolic blood pressure, diastolic blood pressure, baseline strain measurements, troponin, atherosclerotic cardiovascular disease score, albumin, albuminuria, ankle-brachial index
Clinical Markers	Angina, left ventricular diastolic dysfunction, new murmur, acute pericarditis, cardiac tamponade
Medical History	Pre-existing cardiovascular disease, heart failure, coronary artery disease, myocardial infarction, valvular heart disease, cancer therapy-related cardiac dysfunction, stroke or transient ischemic attack, venous thromboembolism, Raynaud’s, cerebrovascular disease, thromboembolic disease, cardiomyopathy, arrhythmia, cardiothoracic surgery, atherosclerosis, retinopathy, neuropathy, pregnancy-associated disorders, human immunodeficiency virus, liver disease, systolic anterior motion
Family History	Early cardiovascular disease, hypercholesterolemia, premature coronary artery disease
Cancer Type	Mediastinal tumors, cardiac metastasis, primary tumors of the heart
Imaging	Echocardiography, 2-dimensional echocardiography, 3-dimensional echocardiography, study quality
Timing	Evaluation before, during, or after cancer treatment (> 6 months and < 12 months)
Medications	Metformin, lipid-lowering therapy (Proprotein convertase subtilisin/kexin type 9 inhibitors, statin therapy, fenofibrate), chemotherapy (high/low dose epirubicin, high/low dose doxorubicin, trastuzumab, 5-Fluorouracil/capecitabine, paclitaxel, vascular endothelial growth factor inhibitors, erlotinib, nilotinib, ponatinib, thalidomide, lenalidomide), sequential or combination therapy
Radiation	Location (anterior or left chest, chest, head/neck, abdomen/pelvis), dose (> 2 Gy/day, ≥ 35 Gy, > 30 Gy), lack of shielding during radiation therapy

### Study population

We considered use cases for patients with breast or other cancers treated particularly with pharmacologic or radiation cancer therapies. We included guidelines, expert consensus, and medical society scientific statement recommendations for both short- and long-term cancer survivors, for prevention of cardiovascular disease before, during, and after cancer therapy.

### Study approach

Each selected guideline, expert consensus, or medical society scientific statement was reviewed. Recommendations were reproduced as conditions for query, with use of formulae including an IF THEN ELSE format. Action steps from the recommendations were collated for composite use in the tool.

### Knowledge management

In the small and emerging field of cardio-oncology, we selected all guidelines, expert consensus documents, and medical society scientific statements relevant to the most common cardiotoxicity presentation (i.e., cardiomyopathy) noted in cardio-oncology clinics nationwide [17, 18, 20–22, 31–33], or relevant to the suboptimal medication use highlighted in the cardio-oncology literature [14–16]. This led us to convert 10 crucial guidelines into query phrases and conditions in the software. This number was decreased to 7 after recognizing and removing redundancies or unclear suggestions. The rules for each guideline were placed in a separate Excel spreadsheet. Then all 7 spreadsheets were consolidated into one, with each guideline represented in a separate tab for simplicity. In this way, all 7 guidelines were formatted in 7 tabs in one combined spreadsheet, with an 8th spreadsheet collating the output from the other 7. We reviewed the final product with patient advocates related to our cardio-oncology clinical and research program, as well as study cardiologists, in addition to the hematologists, oncologists, radiation oncologists, and primary care providers that most frequently refer patients for cardiovascular evaluation, with a total of 25 individuals.

### Automatic formulae

In Fig. 3, column A includes the label for the particular guideline recommendation presented in each row of the Excel file. Column B represents the patient characteristics or condition that must be met to fulfil criteria for the guideline recommendation presented in each row. Column C is optional and presents the patient characteristics or condition as T or F states that must be met to fulfil criteria for the guideline recommendation presented in each row. Column D can be entered manually by ancillary staff for small groups of patients in cardio-oncology, or in the future automated for large populations of patients. A number 1 in Column D means the patients’ characteristics fulfil criteria for the guideline recommendation presented in each row. A number 0 in Column D means the patients’ characteristics do not fulfil criteria for the guideline recommendation presented in each row. Column E provides the recommended action that should be pursued if the patients’ characteristics fulfil criteria for the guideline recommendation presented in each row.

In Fig. 4, the patient characteristics that fulfil criteria for the guideline recommendation presented in each row are presented on the left, and the recommended action that should be pursued if the patients’ characteristics fulfil criteria for the guideline recommendation presented in each row are presented on the right. These are automatically populated, based on entries from Column D in Fig. 3. The automated population simply uses a common Excel formula. The *“if then else”* structure of the formula is as follows. In our case, if the data in a particular cell (e.g., a *1* or *0* in cell *D6* representing whether the patients’ characteristics fulfil criteria for the guideline recommendation) in the Excel spreadsheet is equal to a certain parameter (e.g., *D6 = 1* indicating that the patients’ characteristics fulfil criteria for pursuing the guideline recommendation), then specific output is given which in this case (e.g. *B6* representing the posed criteria for the guideline recommendation that fit the patient), or else different output is given (e.g., no output at all which can be represented as *“”*). The formula captures this system in the form of *= IF((Rules!D6 = 1),Rules!B6,””)*, with *Rules!* indicating the spreadsheet where the cells *D6* and *B6* are found. Similarly, the formula *= IF((Rules!D6 = 1),Rules!E6,””)* captures the expectation that if in the *Rules* spreadsheet the patients’ characteristics fulfil criteria for pursuing the guideline recommendation in row *D6* (denoted as *D6 = 1*), then the specific output would be the content of cell *E6*, or else no output is given (represented as *“”*).

## Results

### Rules-based structure for recommendations

A total of 10 guidelines, expert consensus, and medical society scientific statements were initially selected for use in this study. Then 7 were maintained, due to overlap and updates in guidelines collectively among societies (Table 1). For the total of 7 selected guidelines, extensive formulae were used to convert the guideline recommendations into rules and suggested action steps. On average, each rules-based conversion was completed over 5 h, including initial quality cross-checks. Additionally, 5 h were used to combine all of the recommendations rules and action steps into a composite form. Finally, 5 h were used to assess patient fit using guidelines rules and suggest action steps based on multiple use case examples.

### Use case example

**Fig. 2:**
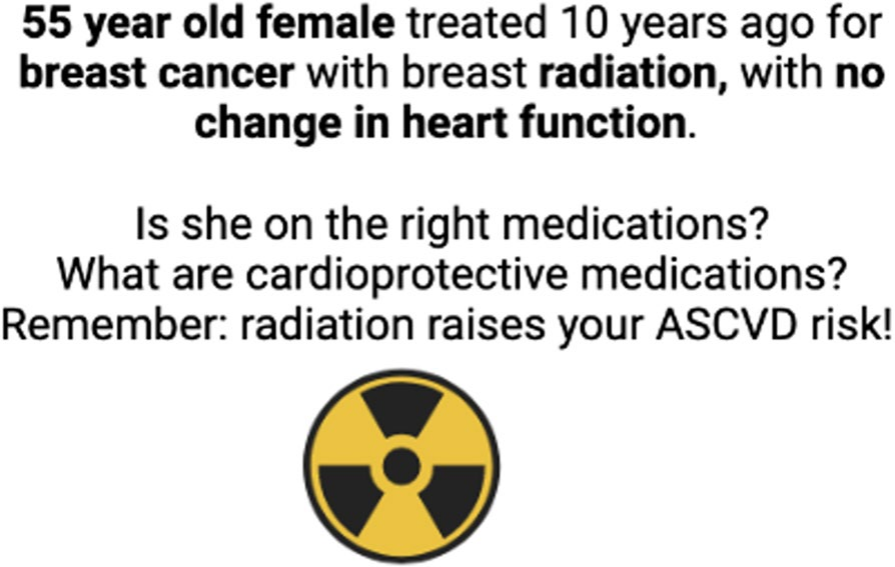
ASCVD = atherosclerotic cardiovascular disease

**Fig. 3:**
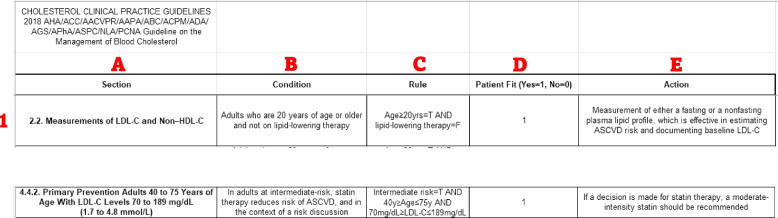
Snapshot of patient fit to particular guideline with focus on cholesterol assessment and statin therapy. ASCVD = atherosclerotic cardiovascular disease; HDL-C = high density lipoprotein cholesterol; LDL-C = low density lipoprotein cholesterol

**Fig. 4:**
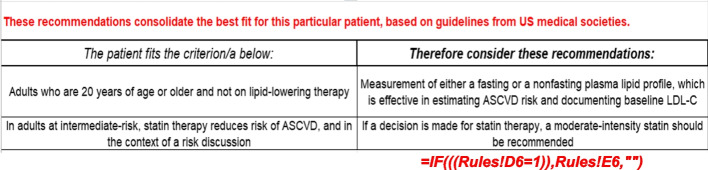
Output customized to preventing ASCVD with focus on cholesterol assessment and statin therapy. ASCVD = atherosclerotic cardiovascular disease; LDL-C = low density lipoprotein cholesterol. The *“if then else”* structured formula *=IF((Rules!D6=1),Rules!E6,””) *shown on the left at the bottom of the figure captures the expectation that if in the *Rules* spreadsheet the patients’ characteristics fulfil criteria for pursuing the guideline recommendation in row *D6* (denoted as *D6=1*) shown in Figure 3, then the specific output would be the content of cell *B6*, or else no output is given (represented as *“”*). The *“if then else”* structured formula *=IF((Rules!D6=1),Rules!E6,“”)* capturesthe expectation that if in the *Rules* spreadsheet the patients’ characteristics fulfil criteria for pursuing the guideline recommendation in row *D6* (denoted as *D6=1*), then the specific output would be the content of cell *E6*, or else no output is given (represented as *“”*)

Our use case example illustrated in Fig. 2 focuses on the prevention of ASCVD in a long-term cancer survivor. The scenario presents a 55 year old female patient who was diagnosed with breast cancer 10 years prior. Her cancer was treated with surgical resection and chest radiation. Given her increased risk of cardiovascular disease as a cancer survivor, relative to the general population, she undergoes cardiovascular assessment with a particular focus on the prevention of ASCVD. Note that her history of radiation can also affect the development of valve disease, pericardial or myocardial disease, arrhythmias, cardiomyopathy, and so on [28]. However, in this use case, the focus is on preventing ASCVD with cholesterol assessment and statin therapy. This use case is highlighted, due to statistically lower rates of statin use in cancer survivors than indicated by guidelines [16]. In this case, statin therapy would be recommended, as shown in Figs. 3 and 4.

### Using the tool in clinical workflow

**Fig. 5:**
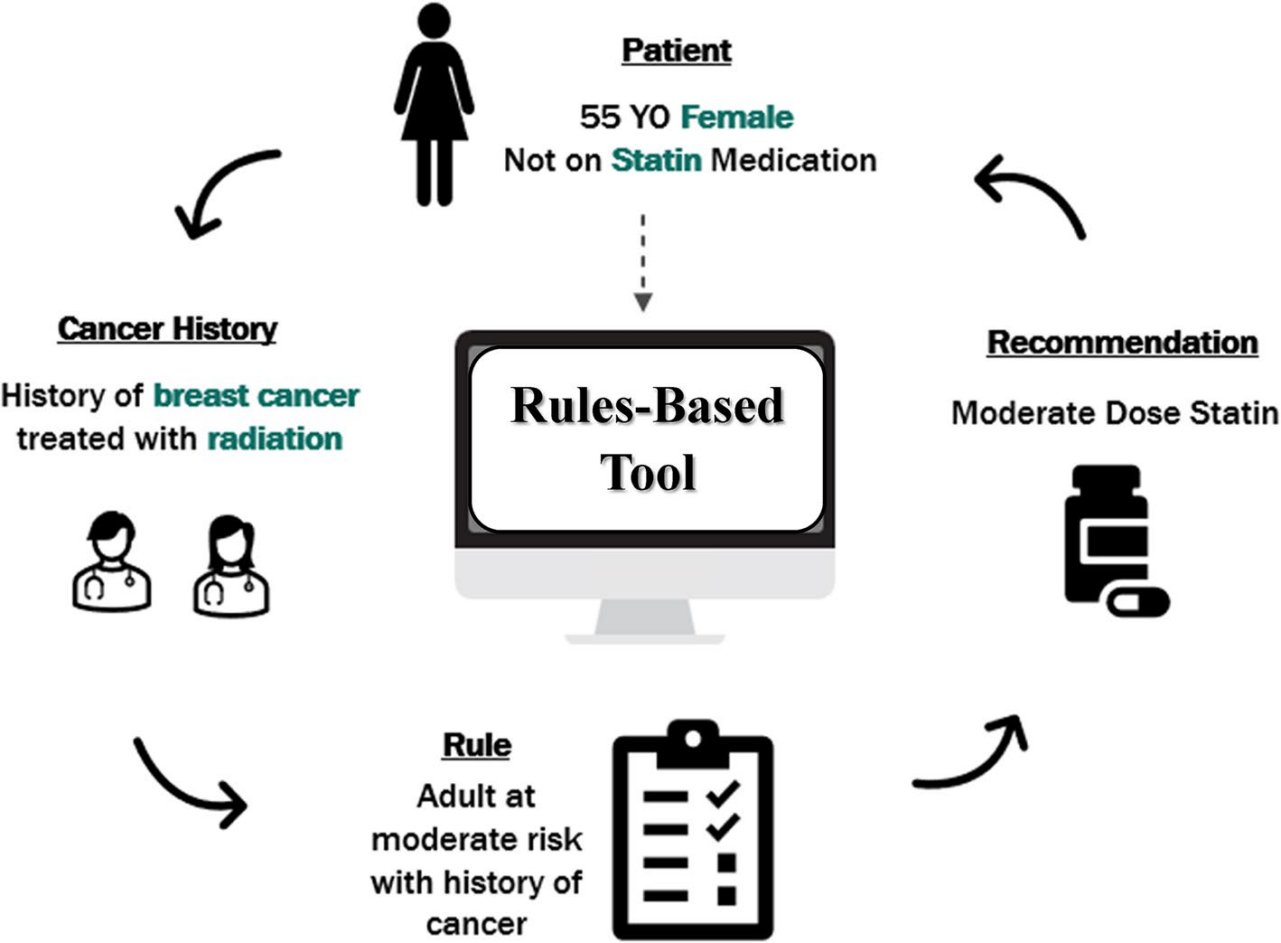
Workflow for using rules-based tool at the point of care for cancer survivors with focus on cholesterol assessment and statin therapy

A sample workflow of using this tool is shown in Fig. 5, with an example focus on the prevention of ASCVD in particular to emphasize the use of statin therapy when clearly indicated by guidelines. In the figure, patient information is identified based on demographics such as age, sex, and specific criteria related to cholesterol assessment and management. The patient’s cancer history and treatment can also be assessed (as in Fig. 2). Based on patient and past medical history characteristics, relevant rules are identified specifically for the patient, with subsequent action steps highlighted. The provider will then be able to utilize this rule-based structure to formulate customized recommendations. The tool is currently placed in the electronic health record and also is available for web-based access in the pending clinical trial (NCT05377320).

## Discussion

A simple rules-based tool was developed to present knowledge assets relevant to point of care cardiovascular disease prevention in cancer survivors. A variety of guidelines were compiled to create a systematic approach to covering various cancer types and factors relating to cardiovascular risks in cancer survivors. Expert consensus and recommendations were converted in the software based on patient characteristics. Formulae were created based on rules and actions from ultimately 7 published recommendations documents. In this way, a tool was developed to facilitate cardiovascular screening and prevention in survivors of various cancers.

Clinicians and patients have limited availability of guidelines for preventing cardiovascular disease in long-term cancer survivors, particularly regarding medication use. Nevertheless, ASCVD guidelines developed for the general population can be extrapolated to at least the minimum care that cancer survivors should receive. These guidelines are included and emphasized in the rules-based tool. The tool can therefore be used in particular to address the care gap in ASCVD risk management in cancer survivors, using guidelines developed for the general population. While ASCVD is highlighted as a special focus, the tool is designed to improve care in managing cardiovascular risk in the various cancer survivor populations.

Statins prevent cardiomyopathy and can be individualized and recommended, especially for patients already on statin therapy or with other indications of starting statin therapy [34]. However, using it specifically for preventing cardiomyopathy is not in the tool. Instead, the tool uses statins to prevent ASCVD, including in individuals with cancer. Additionally, while some studies suggest potentially using statins specifically in patients who have had radiation therapy to the chest [35], this has also not been included in the tool as this has also not been supported in guidelines.

Due to their anti-inflammatory, antioxidant, and cholesterol-lowering properties, statins are frequently used to prevent cardiovascular disease. Statins work by inhibiting hydroxymethyl glutamyl coenzyme A (HMG-CoA). However, they also have a pleiotropic effect by inhibiting small Ras-homologous GTPase, which lowers the inhibition of topoisomerase II and the production of reactive oxygen species [36]. Considering that these two pathways are implicated in cardiotoxicity caused by anthracyclines and trastuzumab, the mechanism of action of statin medications is particularly important in mitigating these effects. This is of special interest to those involved in the burgeoning field of cardio-oncology, a subset of cardiovascular medicine dedicated to preventing and managing the effects of cancer therapy on the cardiovascular system. An expanding body of research is dedicated to cardioprotective measures for cancer patients, but there are no established rules for using statins in this population. A meta-analysis on the safety and efficacy of cardioprotective drugs in chemotherapy-induced cardiotoxicity indicated that cancer patients may not receive evidence-based cardioprotective therapy [37]. The meta-analysis included 33 randomized controlled trials comprising 3,285 patients. Clinical and laboratory cardiac function parameters were assessed, including left ventricular ejection fraction (LVEF), clinical heart failure, troponin levels, and B natriuretic peptide levels [37]. Three of the 33 randomized controlled trials included in the meta-analysis compared statins with a placebo. One of the studies included in the meta-analysis [38] compared atorvastatin with a placebo as prophylactic treatment for patients exposed to anthracycline therapy. Atorvastatin was found to significantly lower the decrease in mean LVEF (*p* < 0.0001) with an insignificant change in the statin group (61.3 ± 7.9% vs. 62.6 ± 9.3%, *p* = 0.144) and a significant decrease in the control group (62.9 ± 7.0% vs. 55.0 ± 9.5%, *p* < 0.0001) [38]. In a second trial [39], statin use in patients receiving anthracycline therapy was compared with participants who received anthracycline treatment but not statin therapy. LVEF in the statin-receiving group was 56.6 ± 1.4% at baseline and 54.1 ± 1.3% six months after initiating anthracycline treatment (*p* = 0.15). In contrast, LVEF in the non-statin group was 57.5 ± 1.4% at baseline and significantly decreased to 52.4 ± 1.2% over a similar six-month anthracycline treatment interval (*p* = 0.0003). When age, sex, DM, HTN, HLD, and cumulative anthracycline received were controlled for, LVEF remained unchanged in participants receiving a statin (+ 1.1 ± 2.6%), while LVEF in those not receiving a statin declined by − 6.5 ± 1.5% (*p* = 0.03). In a third trial [40], the use of rosuvastatin was compared with a placebo demonstrating that the prophylactic use of statin therapy may prevent the development of chemotherapy-induced cardiotoxicity as there was no significant decrease in LVEF compared to baseline in the rosuvastatin group despite a significant reduction in LVEF compared to baseline in the placebo group (intergroup *p* = 0.012). Through the pooling of data in the meta-analysis, a comparison of various single-drug cardioprotective effects was made showing spironolactone to have the greatest significant improvement in LVEF compared to control (MD = 12.80, 95% CI [7.90; 17.70]), followed by enalapril (MD = 7.62, 95% CI [5.31; 9.94]), nebivolol (MD = 7.30, 95% CI [2.39; 12.21]), statin (MD = 6.72, 95% CI [3.58; 9.85]), bisoprolol (MD = 5.72, 95% CI [0.78; 10.66]), perindopril (MD = 5.27, 95% CI [1.75; 8.79]), and carvedilol (MD = 2.54, 95% CI [1.09; 3.99]). Another pooled estimate within the meta-analysis, this time determining improvement in EF compared to control by drug family, showed that statins were associated with the greatest significant improvement (MD = 6.72, 95% CI [3.36; 10.08]). In a study independent of this meta-analysis [41], statin exposure and heart failure risk after receiving anthracycline-based chemotherapy for breast cancer were studied, demonstrating that women exposed to statins had a lower incidence of heart failure hospital presentations after receiving anthracycline-based chemotherapy at 1.2% (95% CI, 0.5-2.6%) compared to 2.9% (95% CI, 1.7-4.6%) in patients not taking statins (*p* = 0.01). Statins have also been shown to reduce major adverse cardiovascular events in the general population and prevent cardiac dysfunction caused by cancer treatment. Ongoing trials such as PREVENT (PREVENTing anthracycline cardiotoxicity with statins), STOP-CA (Statins TO Prevent the Cardiotoxicity from Anthracyclines), and SPARE-HF (Statins for the PrimAry pREvention of Heart Failure in patients receiving anthracyclines) are highly anticipated in this area of study as well [42].

The tool could potentially be used as a clinical decision aid. A clinical decision aid is a tool designed to assist patients and their clinicians in making informed health care decisions, promote patient engagement in the medical care decision-making process, aid clinicians in considering relevant recommendations, and improve patient adherence to their treatment plan [43]. Even though such instruments have been utilized in oncology clinical practice, investigations have revealed a low usage rate [44]. A prevalent barrier to the use of clinical decision aids, according to a study [44], entailed the concern that patients were unable to interpret information from a decision aid. However, when clinicians use clinical decision aids appropriately and integrate them into their practice, patient outcomes improve [45]. In addition, patients who are exposed to clinical decision aids are more likely to engage in decision-making and make decisions of higher quality [46].

Studies also suggest that the use of clinical decision aids can improve patient outcomes [44, 45, 47–53]. Decision aids have been shown to enhance medication adherence and aid in the decision-making process relating to medication use (especially for statin initiation) in the prevention of cardiovascular diseases [47, 50, 54–56]. In the Myocardial Infarction Genes (MI-GENES) trial, a clinical decision aid was developed to determine whether integration of a genetic risk score into the evaluation of coronary heart disease risk lowers low-density lipoprotein cholesterol (LDL-C) levels during clinic visits in the general population. The study found that participants who received genomic risk information in the clinical decision aid group had lower LDL-C levels than those who received conventional risk information without the use of a clinical decision aid. Furthermore, participants who received genomic risk information in the clinical decision aid group were more likely to have cardioprotective medication (i.e., statin therapy) initiated. The findings of the MI-GENES trial demonstrate that the integration of a clinical decision aid for assessing and communicating risk can aid in preventing cardiovascular disease in patients [50]. Based on studies such as these, clinical decision aids could improve cardiovascular disease prevention among patients when used to determine and disclose risk.

There are no widely available tools that facilitates the application of recommendations for the prevention of cardiovascular diseases in cancer survivors. Furthermore, the cholesterol management guidelines published and endorsed by the American Heart Association, American College of Cardiology, and other medical societies encourage a multifaceted approach to the application of these guidelines [30]. Our tool can facilitate the application of these guidelines (Fig. 5), in such a multifaceted approach. To this end, the tool is being prepared for use in an upcoming clinical trial (NCT05377320) to assess outcomes related to use of the tool in clinical practice. In the study, the intervention arm will have early access to the use of the tool. The knowledge assets in the tool will be applied at the point-of-care, to guide patient options for cardiovascular medication and imaging surveillance choices. Patient and clinician satisfaction with the use of the tool in shared decision-making conversations will also be evaluated.

Accuracy and quality control are ensured in real time by manual entry of data into the clinical decision aid in its present form. Future versions of the aid that automate data entry and analysis in the aid can undergo appropriate rigorous scientific and biostatistical methodology to importantly validate a more automated version of the tool. This would also facilitate ultimately incorporating the currently web-based tool into the electronic health record more directly. Optimally, the aid would also be integrated with methods such as flowsheets, smart text/phrases, and autogenerated documentation in care pathways. This would be consistent with other successful studies on the incorporation of rules-based clinical decision aids in clinical practice [57]. Nudges and alerts can also be integrated, to encourage the use of the clinical decision aid for relevant patients. It would be important to assess alert fatigue (number of alerts), behavior influence (number of clinical decision aid access counts), and task completion (number of cardio-oncology referrals via alerts), which are three common metrics used for analyzing the impact of alerts and nudges in electronic health records [58].

It is important to note that the tool will need to be periodically and continually updated whenever new guidelines relevant to the point of care in cardio-oncology are published with substantial new and different recommendations. In light of this, the tool already encourages users to consider the implications and applications from findings in the past year. This includes data from the CAROLE study, in which women received an elective chest x-ray, electrocardiogram, and transthoracic echocardiogram 10 years after treatment for breast cancer. Women had undergone surgical resection only, radiation therapy, and/or various pharmacologic cancer therapies. The study discovered undiagnosed cardiovascular diseases and suggested the use of such screening tools at this timepoint for women treated for breast cancer [23]. In addition, we identified no clearly delineated guidelines specific to racial and ethnic minorities and encouraged shared decision making and individualization across guidelines documents.

## Conclusion

Evidence of limited application of cardiovascular risk and prevention guidelines in cancer survivors requires urgent intervention for this vulnerable population. Consequently, in this brief study we reviewed current guidelines, expert recommendations, and medical society recommendations based on specific patient demographics to create a concise, systematic rules-based tool. The tool was fashioned to use formulae and queries in order to compute suggestions for clinicians based on patient and cancer therapy associated factors to potentially improve future cardiovascular care. In particular, a focus was placed on addressing care gaps related to the prevention of ASCVD with initiation of statin therapy when indicated. In cardio-oncology, morbidity and mortality may potentially be curbed by facilitating the application of crucial guidelines relevant to cancer survivors to preempt and prevent cardiovascular outcomes. Our tool is being made available for this purpose in a pending clinical trial (NCT05377320). Effort will be needed to ensure equity of applying these guidelines especially for racial and ethnic minorities.

## Data Availability

Data can be provided upon request.
